# Chronic Stress-Related Neural Activity Associates With Subclinical Cardiovascular Disease in a Community-Based Cohort: Data From the Washington, D.C. Cardiovascular Health and Needs Assessment

**DOI:** 10.3389/fcvm.2021.599341

**Published:** 2021-03-10

**Authors:** Tiffany M. Powell-Wiley, Amit K. Dey, Joshua P. Rivers, Abhishek Chaturvedi, Marcus R. Andrews, Joniqua N. Ceasar, Sophie E. Claudel, Valerie M. Mitchell, Colby Ayers, Kosuke Tamura, Cristhian A. Gutierrez-Huerta, Heather L. Teague, Steffen G. Oeser, Aditya Goyal, Aditya A. Joshi, Billy S. Collins, Yvonne Baumer, Stephanie T. Chung, Anne E. Sumner, Martin P. Playford, Ahmed Tawakol, Nehal N. Mehta

**Affiliations:** ^1^Social Determinants of Obesity and Cardiovascular Risk Laboratory, National Heart, Lung, and Blood Institute (NHLBI), National Institutes of Health, Bethesda, MD, United States; ^2^Intramural Research Program, National Institute on Minority Health and Health Disparities (NIMHD), National Institutes of Health, Bethesda, MD, United States; ^3^Section of Inflammation and Cardiometabolic Diseases, National Heart, Lung, and Blood Institute (NHLBI), National Institutes of Health, Bethesda, MD, United States; ^4^Division of Cardiology, UT Southwestern Medical Center, Dallas, TX, United States; ^5^Section on Ethnicity and Health, Diabetes Endocrinology and Obesity Branch, National Institute of Diabetes and Digestive and Kidney Diseases (NIDDK), National Institutes of Health, Bethesda, MD, United States; ^6^Cardiology Division, Massachusetts General Hospital and Harvard Medical School, Boston, MA, United States; ^7^Cardiovascular Imaging Research Center, Massachusetts General Hospital and Harvard Medical School, Boston, MA, United States

**Keywords:** stress, inflammation, race and ethnicity, community, atherosclerosis, cardiovascular disease, cardiovascular risk, amygdalar activity

## Abstract

**Background:** Psychosocial stress correlates with cardiovascular (CV) events; however, associations between physiologic measures of stressors and CVD remain incompletely understood, especially in racial/ethnic minority populations in resource-limited neighborhoods. We examined associations between chronic stress-related neural activity, measured by amygdalar ^18^Fluorodeoxyglucose (^18^FDG) uptake, and aortic vascular FDG uptake (arterial inflammation measure) in a community-based cohort.

**Methods:** Forty participants from the Washington, DC CV Health and Needs Assessment (DC-CHNA), a study of a predominantly African-American population in resource-limited urban areas and 25 healthy volunteers underwent detailed phenotyping, including ^18^FDG PET/CT for assessing amygdalar activity (AmygA), vascular FDG uptake, and hematopoietic (leukopoietic) tissue activity. Mediation analysis was used to test whether the link between AmygA and vascular FDG uptake was mediated by hematopoietic activity.

**Results:** AmygA (1.11 ± 0.09 vs. 1.05 ± 0.09, *p* = 0.004) and vascular FDG uptake (1.63 ± 0.22 vs. 1.55 ± 0.17, *p* = 0.05) were greater in the DC-CHNA cohort compared to volunteers. Within the DC-CHNA cohort, AmygA associated with vascular FDG uptake after adjustment for Framingham score and body mass index (β = 0.41, *p* = 0.015). The AmygA and aortic vascular FDG uptake relationship was in part mediated by splenic (20.2%) and bone marrow (11.8%) activity.

**Conclusions:** AmygA, or chronic stress-related neural activity, associates with subclinical CVD risk in a community-based cohort. This may in part be mediated by the hematopoietic system. Our findings of this hypothesis-generating study are suggestive of a potential relationship between chronic stress-related neural activity and subclinical CVD in an African American community-based population. Taken together, these findings suggest a potential mechanism by which chronic psychosocial stress, such as stressors that can be experienced in adverse social conditions, promotes greater cardiovascular risk amongst resource-limited, community-based populations most impacted by cardiovascular health disparities. However, larger prospective studies examining these findings in other racially and ethnically diverse populations are necessary to confirm and extend these findings.

## Introduction

Chronic psychosocial stress, both at the individual and environmental level, is known to be associated with cardiovascular (CV) risk (1). Specifically, chronic psychological stress has been associated with higher risk of obesity (2), hypertension (3), diabetes (4, 5), and poor CV outcomes, including myocardial infarction (6–8), and stroke (9). Perceived adverse neighborhood environment conditions as a type of chronic psychosocial stress has also been associated with obesity (2, 10, 11) and other CV risk factors (11). However, the biological plausibility of a connection between psychological stress and cardiovascular disease in an at risk, community-based cohort remains to be fully explored.

Efforts to elucidate mechanisms by which chronic psychosocial stress may increase CV risk have focused recently on identifying parts of the brain that may be activated by chronic stress (12). The amygdala has been shown to be an important part of the neural network that responds to threatening situations and its activity appears to be heightened in the setting of social stressors related to peer evaluation with associated increases in pro-inflammatory cytokines (13). Recent work has also demonstrated that resting amygdalar activity is associated with worsening aortic vascular inflammation and greater risk for subsequent cardiovascular events (14). Moreover, we have shown that amygdalar activity is positively related to non-calcified coronary artery burden as measured by coronary computed tomography angiography (CCTA) in a chronic inflammatory state (15). These prior studies also found that resting amygdalar activity is associated with greater bone marrow activity in turn driving higher aortic vascular inflammation, suggesting the existence of a “neural-hematopoietic-inflammatory” axis in the development of subclinical cardiovascular disease. Neural responses in the amygdala in the setting of chronic stress likely stimulate hematopoiesis leading to excess production of pro-inflammatory immune cell populations, and subsequently contribute to atherogenesis (16).

Much of the recent work evaluating the relationship between amygdalar activity and cardiovascular disease (CVD) has been done in patients identified in the clinical setting (14) or with known chronic inflammation (15). Additionally, racial/ethnic diversity of prior study populations has been limited. For example, Tawakol et al. provided critical data for the relationships between perceived stress (14) as well as environmental stressors (17, 18) and amygdalar activity in clinical populations; however, the populations in these studies were 90% white or greater, which limits the interpretation of the findings in racial/ethnic minority groups at greatest risk for CVD. Thus, there is a need to focus on diverse, community-dwelling populations, particularly those most impacted by social determinants that promote psychosocial stress and contribute to disparities in CV outcomes (19). Ultimately, work in uncoupling mechanisms by which psychosocial stress leads to CV events in community-based populations with imposed vulnerability may aid in developing targeted interventions that reduce chronic, psychosocial stress and CVD risk. Therefore, we conducted cardiometabolic phenotyping of an at-risk, African-American, community-based population recruited as a part of a community-based participatory research study in Washington, D.C. to examine the relationship between resting amygdalar activity and aortic vascular FDG uptake as measured by ^18^Fluorodeoxyglucose Positron Emission Tomography Computed Tomographic (^18^FDG PET CT) imaging. We hypothesized that amygdalar activity would be greater in the community-based cohort than in healthy volunteers, but differences would be attenuated when accounting for racial/ethnic differences between the populations. Furthermore, we hypothesized that among the community-based cohort, amygdalar activity would be directly associated with aortic vascular FDG uptake beyond traditional cardiovascular risk factors, but this association could be confounded by neighborhood socioeconomic status as a neighborhood social environment stressor (11). Finally, we hypothesized that the relationship between amygdalar activity and aortic vascular FDG uptake would be in part mediated by hematopoietic (leukopoietic) system activity.

## Materials and Methods

### Study Population

Study approval was obtained from the Institutional Review Board (IRB) at National Heart, Lung and Blood Institute (NHLBI), National Institutes of Health (NIH) in accordance with the principles of Declaration of Helsinki. All guidelines for good clinical practice and those set forth by the NIH Radiation Safety Commission and in the Belmont Report (National Commission for the Protection of Human Subjects of Biomedical and Behavioral Research) were followed. All study participants in the cohort provided written informed consent. All the participants and healthy volunteers were adequately compensated.

#### Washington, D.C. CV Health and Needs Assessment Population

The Washington, D.C. CV Health and Needs Assessment (DC-CHNA) was a community-based participatory research-designed, observational study to evaluate CV health factors, neighborhood environment characteristics, and psychosocial variables in a predominantly African-American faith-based population in Washington, D.C. communities at risk for significant CVD (20). As described previously, the DC-CHNA serves as a preliminary step in the development of community-based behavioral change interventions to improve CV health in this community (21). The DC-CHNA was approved by the NHLBI Institutional Review Board (NCT01927783). Informed consent was obtained from DC-CHNA study participants. Participants in the DC-CHNA were recruited to the NIH Clinical Center for additional cardiometabolic phenotyping. Of the 100 DC-CHNA participants, all participants were offered the opportunity to come to the NIH Clinical Center, 58 declined or never responded to the offer, and 42 participants were enrolled into a separate clinical protocol for cardiometabolic testing for those at risk for cardiovascular disease which was approved by the NHLBI Institutional Review Board (NCT01143454). Informed consent for both the DC-CHNA and the cardiometabolic testing protocols was obtained for all the study participants. One participant was unable to undergo ^18^FDG PET/CT imaging due to claustrophobia and one participant was unable to provide amygdala measures due to poor image quality, leaving a final DC-CHNA population of 40 participants. Supplementary Figure 1 demonstrates the recruitment scheme for participants in the DC-CHNA cohort and healthy volunteers.

#### Healthy Volunteer Population

All healthy volunteers were >18 years of age at the time of recruitment and provided written informed consent for testing after a full explanation of the procedures. Healthy volunteers, who were age- and sex-matched to the best of our ability (*N* = 25), were included for comparison with the community-based cohort if they had an estimated glomerular filtration rate > 30 mL/min/1.73 m^2^, no existing CVD, no comorbid condition known to promote CVD or systemic inflammation, such as uncontrolled hypertension, internal malignancy within 5 years or human immunodeficiency virus, no active infection within the past 72 h of baseline, no major surgery within past 3 months, and were not pregnant or lactating.

### Clinical Data and Laboratory Measurements

Demographics, clinical histories, and anthropometric measurements were obtained, and physical examinations were performed by our health-care provider team upon recruitment of the DC-CHNA cohort and the healthy volunteer population. Blood samples were collected after an overnight fast and analyzed for basic chemistry, complete lipid profile, glycosylated hemoglobin, and high sensitivity C-reactive protein at the NIH Clinical Center. Interleukin (IL)-1β, IL-6, IL-8, IL-18, monocyte chemoattractant protein (MCP)-1, and tumor necrosis factor (TNF)-α were measured using a multiplexed ELISA (Meso Scale Diagnostics, Rockville MD, USA), as described previously (22).

### Imaging Measures

Amygdalar activity was measured by corrected standardized uptake value (SUV) from ^18^FDG PET/CT (23). The primary outcome for this study was aortic FDG uptake measured as target-to-background ratio using ^18^FDG PET/CT quantification. Secondary outcomes in the study were hematopoietic system activity in the bone marrow and the spleen measured as standardized uptake values (SUVs) using ^18^FDG PET/CT quantification (14).

#### ^18^Fluorodeoxyglucose Positron Emission Tomography Computed Tomographic Imaging Acquisition

Following an overnight fast for at least 8 h, PET/CT images were obtained ~60 min after administration of 10mCi ^18^FDG (23). PET imaging occurred using a Siemens Biograph mCT PET/CT 64-slice scanner (Siemens Medical Solutions USA, Malvern, PA, USA), acquiring 1.5 mm axial slices of the aorta. Standard bed positions of 3 min each, scanning cranially to caudally, were obtained for each patient from the vertex of the skull to the toes.

#### Amygdalar Activity Measurement

The amygdalae are bilateral parts of the brain's limbic system and are located dorso-medially in the temporal lobe, forming the ventral, superior and medial walls of the inferior horn of the lateral ventricle (14, 15). After identification of amygdalar anatomical location using the above landmarks, a single reader placed right and left 3D-volume regions of interest (ROIs) with a fixed volume (0.5 cm^3^) in the desired area and measured ^18^FDG uptake as SUV using dedicated software (OsiriX MD, Geneva, Switzerland) and previously described methods (14, 15). Amygdalar activity (AmygA) target-to-background ratio (TBR) was calculated by dividing the maximum SUVs in each amygdala by the mean SUVs in ipsilateral temporal lobes for correction of amygdala SUV values. The highest AmygA between the two amygdalae was taken as the primary measure of amygdalar activity (14, 15).

#### Aortic Vascular FDG Uptake Measurement

Aortic vascular FDG uptake was quantified using previously published methods (23). ROIs were placed on 1.5 mm thick axial slices of the aorta from the aortic root through the bifurcation into iliac arteries. ROIs were also placed on 10 continuous slices of the superior vena cava to calculate and correct for background venous activity. Mean and maximum SUVs were generated using dedicated software (OsiriX MD, Geneva, Switzerland). TBR was calculated by dividing the maximum SUV from each slice by the average of mean SUVs in the lumen of superior vena cava. A single aortic TBR value per patient was generated by averaging all individual TBR values.

#### Hematopoietic System Activity Measurement

3D-volume ROIs were placed within individual vertebrae (T1 to L5) and the spleen to measure bone marrow and spleen activity. Total bone marrow activity was reported as the average of the maximum SUVs of the individual vertebrae. Spleen activity was reported as the maximum SUV of the ROI in the spleen for each individual.

### Covariates

Relevant medical history including cardiovascular risk factors, hypertension, diabetes, hyperlipidemia, and smoking history were collected from all study participants enrolled in the DC-CHNA. All participants underwent blood draws to assess lipid levels including total, HDL, and low-density lipoprotein (LDL) cholesterol, glucose, and high-sensitivity C-reactive protein levels. Diabetes, hypertension and hyperlipidemia were defined either by an established diagnosis or by use of glucose-lowering, blood pressure-lowering or lipid-lowering drugs, respectively. Metabolic syndrome was defined as having three or more of the following risk factors: abdominal obesity (waist circumference >101.6 cm for men and >88.9 cm for women); triglycerides ≥150 mg/dl; HDL cholesterol <40 mg/dl for men or <50 mg/dl for women; systolic BP ≥130 mmHg or diastolic BP ≥85 mmHg; or fasting glucose ≥100 mg/dl. Framingham risk score was used to investigate the participant's 10-year risk for cardiovascular disease. Framingham risk score was calculated as previously described using six coronary risk factors including age, gender, TC, HDL-cholesterol, systolic blood pressure, and smoking habits (24).

### Neighborhood Deprivation Index

United States Census Bureau data from the 2010 American Community Survey was used to create a Neighborhood Deprivation Index (NDI) for census tracts in Washington, D.C. and Maryland, as previously described (25). Briefly, key variables were identified via principal axis factoring with oblique rotation (minimum loading score 0.40; minimum eigenvalue 1). The broad variable categories included: income, wealth, education, employment/occupation, and housing conditions. Each factor was required to have Cronbach's alpha ≥0.70 for maintain internal consistency. The z-standardized neighborhood variables were summed to create a summary NDI score, with a higher score representing a more deprived neighborhood.

### Statistical Analysis

Summary statistics were generated and expressed as mean with standard deviation for parametric variables and median with interquartile range for non-parametric continuous variables. Categorical variables were recorded as frequencies and percentages. Normality was evaluated using skewness, kurtosis, and histogram plots. Intergroup differences between the DC-CNHA and healthy volunteer cohorts were assessed using Student's *t*-test or the Mann-Whitney *U*-test, as appropriate. Dichotomous variable comparisons were conducted using Pearson's chi-square test. Linear regression modeling was used to examine intergroup differences adjusted for sociodemographics, including race/ethnicity. Multivariable linear regression analyses were used to evaluate the association of AmygA with aortic vascular FDG uptake in the DC-CHNA cohort. We also performed mediation analyses using structural equation modeling in our nested adjusted model to calculate the association and incremental value of hematopoietic system activity on the total effect of AmygA on aortic vascular FDG uptake. STATA 12 (StataCorp, College Station, TX, USA) was used for all analyses. *P* ≤ 0.05 were considered statistically significant. The STATA 12 codes used for this study are displayed in Supplementary Figure 2.

## Results

### Baseline Characteristics

The DC-CHNA cohort consisted of 40 African American participants who were middle aged and predominantly female (*n* = 36, 90%). Compared to the healthy volunteer population, the DC-CHNA cohort was older (mean age of 60 ± 11 vs. 40 ± 17 years, *p* < 0.001), more likely to be African American (100 vs. 8%, *p* < 0.001), less likely to be male (10 vs. 32%, *p* = 0.03) and more likely to have hypertension (68 vs. 16%, *p* < 0.001), hyperlipidemia (68 vs. 24%, *p* = 0.001), and metabolic syndrome (65 vs. 9%, *p* < 0.001) (Table 1). The DC-CHNA population was also more likely to be obese (body mass index: 33 ± 7 vs. 24 ± 5, *p* < 0.001), have higher values for systolic blood pressure (132.4 ± 15.6 vs. 109.2 ± 11.3 mmHg, *p* < 0.001), high sensitivity C-reactive protein [3.0 (1.1–4.3) vs. 1.0 (0.6–2.2) g/L, *p* = 0.002], hemoglobin A1c (5.8 ± 0.9 vs. 5.4 ± 0.9, *p* = 0.02), and Framingham risk score [8.6 (4.1–15.9) vs. 1.6 (1.3–1.9), *p* < 0.001] than the healthy volunteers. Additionally, AmygA (1.11 ± 0.09 vs. 1.05 ± 0.09, *p* = 0.004) were greater in the DC-CHNA cohort compared to the healthy volunteers (Table 1). The higher AmygA among the DC CHNA was attenuated when the intergroup differences were adjusted for race/ethnicity (β = 0.42, *p* = 0.06) and no longer significant when adjusted for race/ethnicity, Framingham risk score, and BMI (β = 0.42, *p* = 0.11).

**Table 1:** Baseline characteristics of DC-CHNA cohort and healthy volunteers, 2014–2017.

**Demographics and medical history**	**DC-CHNA cohort (*n* = 40)^a^**	**Healthy volunteers (*n* = 25)^a^**	***p*-value**
Age, years	60 ± 11	40 ± 17	**<0.001^d,f^**
Males, N (%)	4 (10)	8 (32)	**0.03^b^**
Race, African American, N (%)	40 (100)	2 (8)	**<0.001^d,f^**
Body mass index, kg/m^2^	33 ± 7	24 ± 5	**<0.001^d,f^**
Current smoker, N (%)	4 (10)	2 (8)	0.79
Hypertension, N (%)	27 (68)	4 (16)	**<0.001^d,f^**
Diabetes mellitus, N (%)	8 (21)	2 (8)	0.18
Metabolic syndrome, N (%)	26 (65)	2 (9)	**<0.001^d,f^**
Hyperlipidemia, N (%)	26 (68)	6 (24)	**0.001^c,f^**
Statin treatment, N (%)	14 (36)	2 (8)	**0.01^b^**
**Clinical and laboratory values**			
Systolic blood pressure, mmHg	132.4 ± 15.6	109.2 ± 11.3	**<0.001^d,f^**
Diastolic blood pressure, mmHg	73.5 ± 10.9	70.1 ± 6.6	0.09
Total cholesterol, mg/dL	198.2 ± 40.2	181.5 ± 33.3	**0.04^b^**
HDL cholesterol, mg/dL	58.9 ± 20.1	63.6 ± 16.8	0.17
LDL cholesterol, mg/dL	111.5 ± 35.2	99.4 ± 30.7	0.08
Triglycerides, mg/dL	82.2 ± 26.5	92.7 ± 41.7	0.11
High sensitivity c-reactive protein, mg/L	3.0 (1.1–4.3)	1.0 (0.6–2.2)	**0.002^c^**
Hemoglobin A1c, %	5.8 ± 0.9	5.4 ± 0.4	**0.02^b^**
Framingham risk score	8.6 (4.1–15.9)	1.6 (1.3–1.9)	**<0.001^d,f^**
**Brain and vascular FDG PET/CT imaging**			
Amygdalar FDG uptake (TBR^||^)	1.11 ± 0.09	1.05 ± 0.09	**0.004^c^**
Aortic vascular FDG uptake (TBR^e^)	1.63 ± 0.22	1.55 ± 0.17	**0.05^b^**
**Cytokine characterization**			
Interleukin 1 Beta, pg/mL	0.16 (0.12–0.22)	0.06 (0.04–0.12)	**0.02^b^**
Interleukin 6, pg/mL	1.06 (0.82–1.60)	0.69 (0.45–1.07)	**0.056**
Interleukin 8, pg/mL	3.70 (2.41–4.77)	3.19 (2.00–4.10)	0.34
Interleukin 18, pg/mL	384.37 (273.78–503.90)	392.64 (158.60–474.75)	0.41
Monocyte chemoattractant protein 1, pg/mL	147.74 (118.43–167.25)	106.32 (99.13–138.88)	**0.02^b^**
Tumor necrosis factor alpha, pg/mL	1.52 (1.31–1.84)	0.99 (0.59–1.27)	**<0.001^d,f^**
Interferon gamma, pg/mL	5.03 (3.00–9.51)	6.68 (2.92–8.16)	0.99

a *Values reported in the table as Mean ± SD or Median (IQR) for continuous data and N (%) for categorical data. P ≤ 0.05 deemed significant. P-values were calculated by using student's t-test or Mann-Whitney U-test for continuous variables and Pearson's chi-squared test for categorical variables. Bold font indicates significance*.

b *P ≤ 0.05*,

c *P < 0.01*,

d *P < 0.001*,

e *TBR, target-to-background ratio*,

f *significance after adjustment for multi-variable comparison (p < 0.0018)*.

### Biomarkers of Subclinical Inflammation and Chronic Stress

Individuals in the DC-CHNA had higher Interleukin (IL)-1β [0.16 (0.12–0.22) vs. 0.06 (0.04–0.12) pg/ml, *p* = 0.02], monocyte chemoattractant protein (MCP)-I [147.74 (118.43–167.25) vs. 106.32 (99.13–138.88), *p* = 0.02] and tumor necrosis factor (TNF)-α [1.52 (1.31–1.84) vs. 0.99 (0.59–1.27), *p* < 0.001] when compared to healthy volunteers (Table 1). IL-6 levels trended toward being different as well [1.06 (0.82–1.60) vs. 0.69 (0.45–1.07), *p* = 0.056]. The number of clinical and imaging variables and biomarkers that were statistically different (*p* < 0.05) between the DC-CHNA cohort and healthy volunteers was > 5% of the comparisons in Table 1, suggesting that the differences identified between the two cohorts were not by chance alone. However, when accounting for multiple comparisons between all variables displayed in Table 1 (28 comparisons: *p*-value adjustment <0.0018 for significance) the following variables remain statistically significant: Age, Race/Ethnicity, BMI, Hypertension, Metabolic Syndrome, Hyperlipidemia, Systolic blood pressure, FRS, and TNFα levels.

### Associations Between Amygdalar Activity, Hematopoietic Tissue Activity and Aortic FDG Uptake

Unadjusted regression modeling exploring the relationship between AmygA and aortic vascular FDG uptake in the DC-CHNA cohort indicated a 0.34-unit increase in aortic vascular FDG uptake for every one-unit increase in AmygA (Table 2). This relationship remained significant after adjusting for traditional cardiovascular risk factors such as Framingham risk score and body mass index (β = 0.41, *p* = 0.015). When adjusting for NDI as a measure of neighborhood-level socioeconomic stress and a potential confounder, the association between AmygA and aortic vascular FDG uptake was no longer statistically significant (β = 0.23, *p* = 0.12) (Table 2).

**Table 2:** Regression analyses between amygdalar activity and aortic vascular FDG uptake in DC-CHNA cohort, 2014–2017.

**Models (*N* = 40)^a^**	**Standardized beta^b^ (*p*)**
Unadjusted	0.34 (0.04)^c^
Adjusted for framingham risk score	0.39 (0.02)^c^
Adjusted for framingham risk score and body mass index	0.41 (0.02)^c^
Adjusted for framingham risk score, body mass index, and NDI^d^	0.23 (0.12)

a *Cohort included in modeling with complete data (N = 40)*.

b *All values are reported as Standardized beta coefficient(β), (p) values*.

c *P ≤ 0.05*.

d *NDI – neighborhood deprivation index – measure of neighborhood-level socioeconomic status based on 2010 United States Census data*.

**Figure 1:**
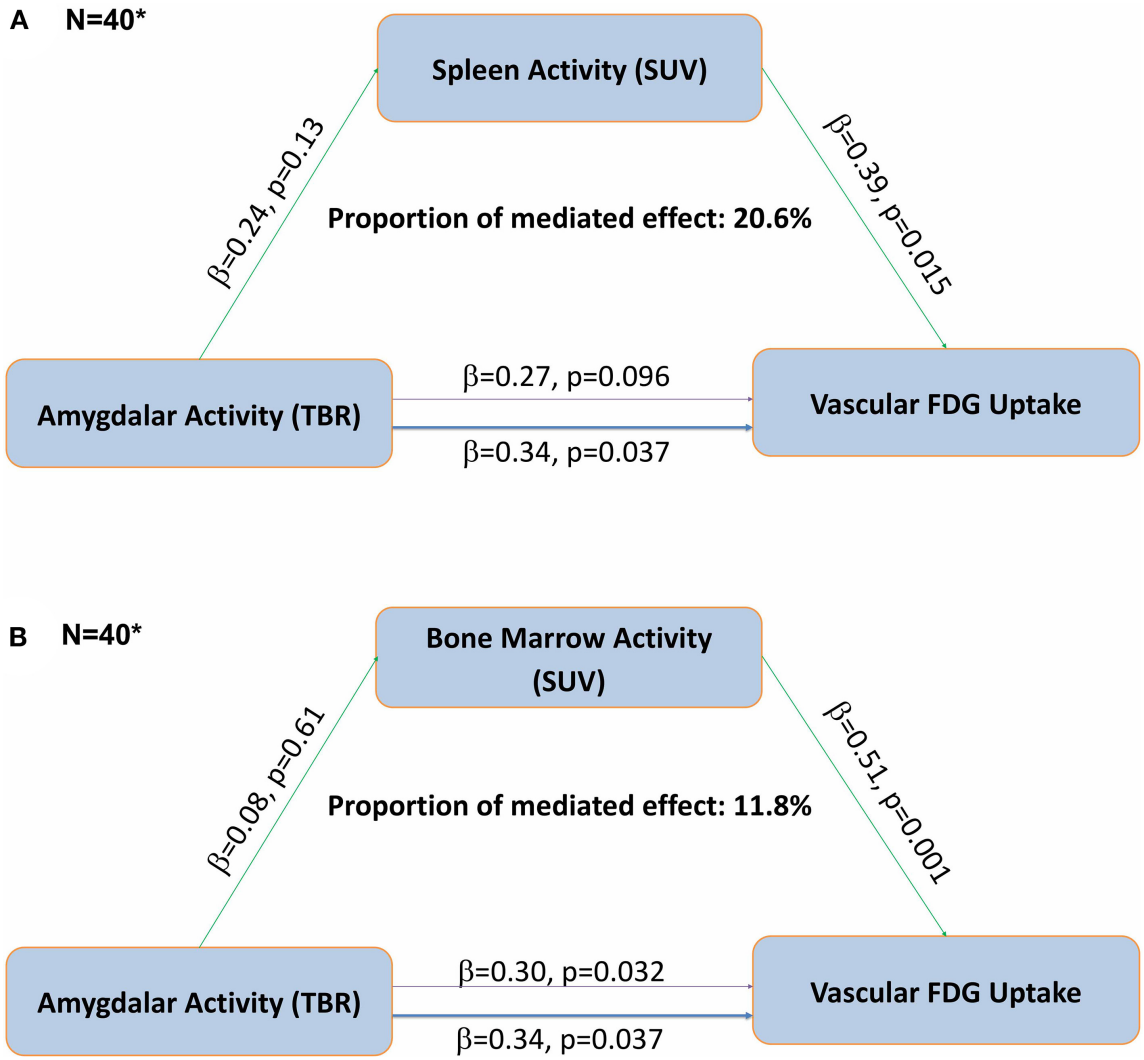
Fully adjusted mediation analyses demonstrate that the association between amygdalar activity and aortic vascular FDG uptake is partially mediated by hematopoietic tissue activity in DC-CHNA Cohort, 2014–2017. **(A)** Mediation analyses demonstrate an indirect effect of splenic activity **(A)** and bone marrow activity **(B)** on the relationship between resting amygdalar and aortic vascular FDG uptake in DC-CHNA Cohort, 2015–2017. **(B)** Mediation analyses demonstrate an indirect effect of bone marrow activity on the relationship between resting amygdalar and aortic vascular FDG uptake in DC-CHNA Cohort, 2015–2017. (*Analyses completed with data from DC-CHNA population (*N* = 40). Thinner purple line represents mediated effect between Amygdalar Activity and Vascular FDG uptake. ^a^TBR, tissue-to-background ratio; ^b^SUV, standardized uptake value).

Regression modeling that examined the relationships between hematopoietic tissue activity and aortic vascular FDG uptake revealed that for every unit increase in bone marrow activity, there was a 0.51-unit increase in aortic FDG uptake (*p* = 0.001). For every one-unit increase in spleen activity, there was a 0.39-unit increase in aortic FDG uptake (*p* = 0.015) (Table 3). Furthermore, in mediation analyses, we found that splenic activity partially mediated the association between AmygA and aortic vascular FDG uptake, accounting for 20.6% of the relationship (Figure 1A). We also demonstrated that bone marrow activity accounted for 11.8% of the relationship between AmygA and aortic vascular FDG uptake (Figure 1B).

**Table 3:** Regression analyses demonstrate direct association between hematopoietic tissue activity and aortic vascular FDG uptake in DC-CNHA cohort, 2014–2017.

**Parameter (*N* = 40)**	**Aortic VI, standardized beta^a^ (*p*)**
Bone marrow activity	0.51 (0.001)^c^
Spleen activity	0.39 (0.02)^b^
Liver activity	0.48 (0.002)^c^

a *All values are reported as Standardized beta coefficient (β), (p) values*.

b *P ≤ 0.05*,

c *P < 0.01*.

## Discussion

In a community-based cohort from resource-limited urban areas of Washington, D.C., we demonstrated that AmygA, as measured by ^18^FDG PET/CT, was higher as compared to healthy volunteers; these differences appeared related to racial/ethnic differences between the populations and differences in risk factor profiles. We also found that AmygA was positively associated with aortic vascular FDG uptake in the community-based cohort; this relationship was attenuated when accounting for neighborhood socioeconomic level as a potential confounder. Additionally, the relationship between AmygA and aortic vascular FDG uptake appears to be mediated in part by hematopoietic activity, suggesting the existence of the neural-hematopoietic-inflammatory axis in this African American, community-based cohort. Taken together, these findings suggest a potential mechanism by which chronic psychosocial stress promotes greater cardiovascular risk amongst resource-limited, community-based populations most impacted by cardiovascular health disparities.

### Amygdalar Activity Is Associated With Aortic Vascular FDG Uptake in a Community-Dwelling Population Living in Under-Resourced Urban Areas

Our findings provide additional evidence that AmygA is linked to aortic vascular FDG uptake, a known marker of cardiovascular risk, and may indicate how chronic stressors worsen cardiovascular outcomes in under-resourced populations. This is one of the first studies to assess the relationship between neural networks associated with chronic stress and cardiovascular risk in a racial/ethnic minority, community-based cohort as compared to a patient population with limited racial/ethnic diversity (14, 17, 18) or with chronic inflammation including psoriasis (15). Our findings highlight that differences in the racial/ethnic composition of the DC-CHNA cohort as compared to the healthy volunteers may explain AmygA differences between the two groups. Moreover, these results support a need to better understand the cardiovascular impact of chronic stressors across the life course that are unique to racial/ethnic minority populations from resource-limited communities (26). Racial/ethnic minority cohorts in under-resourced communities likely experience both individual- and neighborhood-level chronic stressors including adverse life events (27, 28), discrimination (29, 30), financial hardships, limited safety, crime (31), and residential segregation (32, 33), that likely act cumulatively to contribute to racial/ethnic disparities in cardiovascular health (34). These findings expand this body of literature on chronic stress and cardiovascular health in underserved populations to provide potential mechanistic evidence of a link between chronic stress-related neural activity and subclinical vascular disease (35–37). Clark et al. recently demonstrated that self-reported social discrimination, as measured by the well-validated Everyday Discrimination Scale, was associated with greater spontaneous AmygA in a diverse population, identifying social discrimination as a source of chronic stress that can impact neural activity (38).

Our exploratory models incorporating neighborhood socioeconomic status (as measured by U.S. Census derived NDI) indicate that NDI may confound the relationship between AmygA and aortic vascular FDG uptake in the community-based cohort. This suggests that both AmygA and aortic vascular FDG uptake may potentially be impacted by adverse neighborhood environment conditions as a source of chronic stress. Although not comprehensively explored in our analysis, this framework is supported by prior studies showing a link between physical or social environmental factors, AmygA and arterial inflammation. For instance, Osborne and colleagues showed that transportation noise exposure within one's neighborhood could result in CV events due to amgydalar activation and subsequent arterial inflammation (18). We also demonstrated that pro-inflammatory cytokines were elevated in our community-based cohort as compared to the healthy volunteers, a finding which is supported by the evidence that greater chronic psychosocial stress relates to decreased vagal function, which can activate inflammatory pathways, including IL-1 and TNF-α production (39). Further work should explore specific individual or environmental stressors to identify those most associated with AmygA as a measure of chronic stress-related neural activity.

### Hematopoietic System Activity Mediates Relationship Between Amygdalar Activity and Aortic Vascular FDG Uptake in Community-Based Cohort

Hematopoietic system activity, specifically in the bone marrow and spleen, in part mediated the relationship between AmygA and aortic vascular FDG uptake in our community-based cohort. Thus, this is one of the first studies in a racial/ethnic minority, community-based cohort to suggest the existence of the neural-hematopoietic-inflammatory axis (14–16). These findings are further supported by the evidence that brain centers can activate bone marrow cells to promote arterial hypertension in animal models (40). Moreover, recent work in mice demonstrates that induced chronic stress can lead to a prothrombotic state for those with a genetic predisposition to anxiety and depression, highlighting potential gene-by-environment interactions that lead psychosocial stressors to increase cardiovascular risk (41). Further translational work in humans should explore how immune cell function may influence vascular health in the setting of chronic social stressors.

In addition to better understanding the underlying mechanisms linking neural activity related to chronic stress to hematopoiesis and inflammation, more work is also needed to identify interventions that may be particularly effective in reducing the cardiovascular sequalae of chronic stress. For example, physical activity may be especially effective in modifying cardiovascular reactivity due to chronic stress by increasing the sensitivity and effectiveness of visceral control mechanisms for heart rate and blood pressure (42). However, multi-level approaches to behavioral interventions that address cardiovascular health disparities are particularly important (43, 44). For instance, one may consider combining health behavior changes at the individual level, such as interventions to increase physical activity for stress reduction, with environmental approaches, such as public policy to promote safe communities with equitable resources for healthy behaviors (45).

### Strengths and Limitations

Our study has several limitations. Given the cross-sectional study design, our findings do not establish directionality or causality of the relationship. Moreover, ours is a small observational study, and our findings are hypothesis-generating, but should be repeated in larger, diverse population-based cohorts. Despite our attempts at controlling for confounding covariates, residual confounding also remains a concern. In particular, future studies should examine potential confounding by individual-level socioeconomic status, such as income and education. Conversely, it is possible that our models incorporating NDI were underpowered, rather than suggesting a true confounding effect of NDI on the relationship between AmygA and aortic vascular inflammation. Furthermore, we could not incorporate hard cardiovascular outcomes, and used aortic vascular FDG uptake by ^18^ FDG PET/CT as a surrogate of future cardiovascular events (46). We also acknowledge that our healthy volunteer group was not adequately age- and sex-matched to the DC-CHNA cohort. Thus, our results should be interpreted with caution. Future studies should look at longitudinal interventions that may be particularly effective in reducing the cardiovascular sequalae of chronic stress in at-risk populations.

### Conclusions

In conclusion, our findings suggest that AmygA is a valid measure of chronic stress-related neural activity in a resource-limited, urban community-based cohort. Furthermore, chronic stress-related neural activity as assessed by AmygA was elevated in this at-risk population when compared to healthy volunteers and associated with aortic vascular FDG uptake, mediated in part by hematopoietic activity in the spleen and bone marrow. Future work should focus on elucidating physiologic pathways that connect chronic stress-related neural activity with aortic vascular FDG uptake and may serve as targets in interventions to reduce cardiovascular risk in populations most vulnerable to chronic stress.

## Data Availability Statement

The raw data supporting the conclusions of this article will be made available by the authors, without undue reservation.

## Ethics Statement

The studies involving human participants were reviewed and approved by Institutional Review Board (IRB) at National Heart, Lung and Blood Institute (NHLBI), National Institutes of Health (NIH). The patients/participants provided their written informed consent to participate in this study.

## Author Contributions

TP-W and NM conceptually designed and supervised the study. TP-W, JC, YB, and MA drafted the manuscript and figures. TP-W, AD, SEC, CG-H, CA, MA, and JC performed statistical analysis and modeling. AD, JR, AC, AG, and AJ analyzed FDG/PET-CT imaging. MP performed cytokine measurements. VM and BC helped recruiting the study participants. YB, KT, HT, STC, AS, SO, and AT as well as all other authors critically reviewed the manuscript. All authors contributed to the article and approved the submitted version.

## Conflict of Interest

NM is a full-time US government employee and has served as a consultant for Amgen, Eli Lilly, and Leo Pharma receiving grants/other payments; as a principal investigator and/or investigator for AbbVie, Celgene, Janssen Pharmaceuticals, Inc, and Novartis receiving grants and/or research funding and as a principal investigator for the National Institute of Health receiving grants and/or research funding. The remaining authors declare that the research was conducted in the absence of any commercial or financial relationships that could be construed as a potential conflict of interest.

## Supplementary Material

Supplementary Figure 1 Recruitment scheme for DC-CHNA Cohort and Healthy Volunteers. Recruitment Scheme for DC-CHNA Cohort and Healthy Volunteer Populations, 2014–2017.

Supplementary Figure 2 Coding scheme used for statistical analysis in STATA12 software.

## Abbreviations

^18^FDG: ^18^Fluorodeoxyglucose
^18^FDG PET CT: ^18^FDG Positron Emission Tomography Computed Tomography
AmygA: amygdalar activity
BMI: body mass index
BP: blood pressure
CCTA: coronary computed tomography angiography
CV: cardiovascular
CVD: cardiovascular disease
DC-CHNA: Washington D.C. CV Health and Needs Assessment
HDL: high-density lipoprotein
HPA: hypothalamic-pituitary-adrenal
HgbA1c: glycosylated hemoglobin
IL: Interleukin
LDL: low-density lipoprotein
MCP: monocyte chemoattractant protein
NHLBI: National Heart, Lung, and Blood Institute
NIH: National Institutes of Health
ROI: regions of interest
SUV: standardized uptake value
TBR: target to background ratio
TNF: tumor necrosis factor.
