# Supplementary figures and images for: Chronic Stress-Related Neural Activity Associates With Subclinical Cardiovascular Disease in a Community-Based Cohort: Data From the Washington, D.C. Cardiovascular Health and Needs Assessment
Source: Front Cardiovasc Med. 2021 Mar 10;8:599341. doi: 10.3389/fcvm.2021.599341 (PMC7988194; doi:10.3389/fcvm.2021.599341)

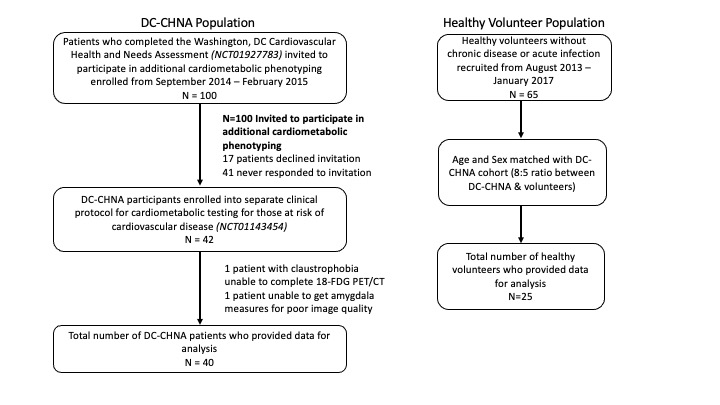

Supplement: Supplementary Figure 1 — Recruitment scheme for DC-CHNA Cohort and Healthy Volunteers. Recruitment Scheme for DC-CHNA Cohort and Healthy Volunteer Populations, 2014–2017. [file Image_1.jpg]

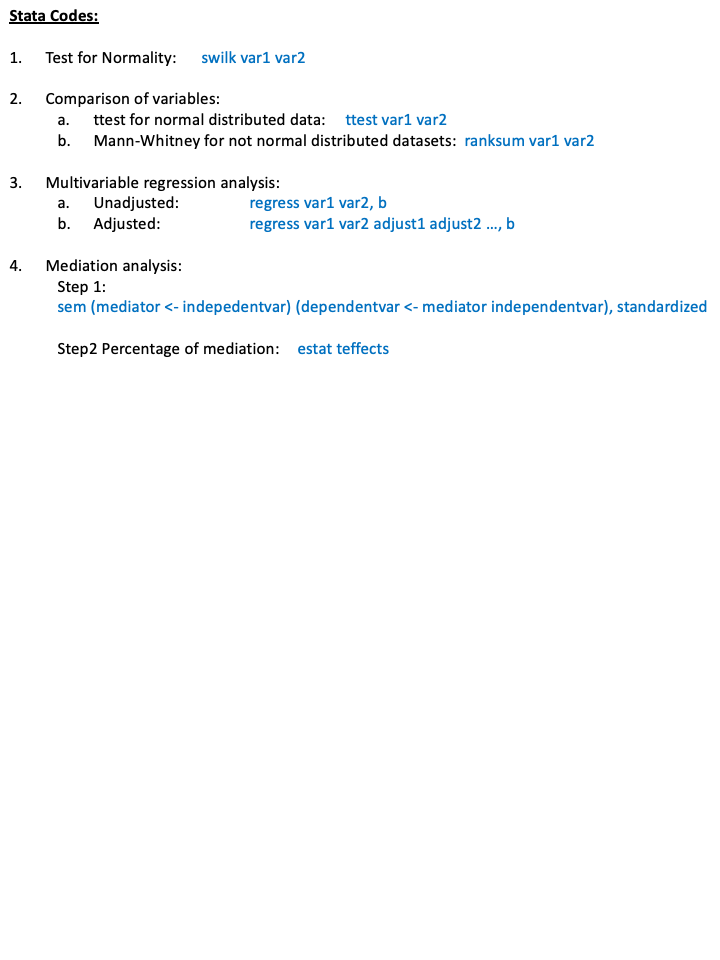

Supplement: Supplementary Figure 2 — Coding scheme used for statistical analysis in STATA12 software. [file Image_2.tiff]
